# Blood Transfusions are Associated With Prolonged Mechanical Ventilation Following Cardiac Surgery in Neonates

**DOI:** 10.31083/RCM36566

**Published:** 2025-06-26

**Authors:** Yansong Zuo, Han Zhang, Lizhi Lv, Gang Li, Ju Zhao, Qiang Wang

**Affiliations:** ^1^Department of Pediatric Cardiac Center, Beijing Anzhen Hospital, Capital Medical University, 100029 Beijing, China; ^2^Department of Cardiopulmonary Bypass, Beijing Anzhen Hospital, Capital Medical University, 100029 Beijing, China

**Keywords:** blood transfusion, neonate, cardiac surgery, mechanical ventilation

## Abstract

**Background:**

To investigate the factors that influence blood transfusions after neonatal cardiac surgery and their association with prolonged mechanical ventilation (PMV) to provide a basis for optimizing blood transfusion strategies.

**Methods:**

This study retrospectively analyzed the clinical data of 202 neonates who had undergone cardiac surgery with cardiopulmonary bypass (CPB) in Beijing Anzhen Hospital from 2019 to 2023. Demographic data, preoperative parameters (body weight, hemoglobin, Risk-Adjusted Classification of Congenital Heart Surgery 1 (RACHS-1) score), intraoperative data (CPB time, aortic cross-clamp time, deep hypothermic circulatory arrest (DHCA)), and transfusions of red blood cells (RBCs), fresh frozen plasma (FFP), and platelet concentrate (PC) within 48 hours after surgery were collected. PMV was defined as mechanical ventilation ≥96 hours after surgery. Multivariate logistic regression was used to analyze independent risk factors for PMV, and the dose–response relationship between transfusion volume and PMV was evaluated by restricted cubic splines (RCSs).

**Results:**

Within 48 hours postoperation, 50.00% of patients were transfused with RBCs, 37.62% were transfused with FFP, and 27.72% were transfused with PC. The PMV incidence was 36.63% in patients with lower body weight (odds ratio (OR) = 0.38, 95% confidence interval (CI): 0.20–0.74; *p* = 0.005), lower preoperative hemoglobin (OR = 0.99; 95% CI: 0.97–0.99; *p* = 0.041), and a RACHS-1 score of 4 (OR = 2.56; 95% CI: 1.04–6.27; *p* = 0.040), and RBCs (OR = 2.02; 95% CI: 1.02–4.00; *p* = 0.043), and FFP infusion (OR = 1.98; 95% CI: 1.02–3.85; *p* = 0.043) were independent risk factors. The RCS demonstrated a linear dose–response relationship between the volume of RBCs infused and PMV (*p* nonlinear = 0.668), whereas there was no association for FFP. The duration of intensive care unit (ICU) stay in patients with PMV (14 days vs. 8 days) and the hospitalization (18 days vs. 13 days) were significantly longer (both *p* < 0.001).

**Conclusion:**

Blood transfusion after neonatal cardiac surgery is an important controllable risk factor for the development of PMV, and its risk increases linearly with the volume of RBC transfusion. Future multicenter prospective studies are needed to validate the causal association further.

## 1. Introduction

Although continuous advances in surgical technology and improved perioperative management have led to improvements in neonatal cardiac surgery, surgical treatment of this vulnerable group remains challenging. Recent research suggests that in-hospital mortality rates for neonatal cardiac surgery have been as high as 9.1% in the past decade [1]. Neonatal bloodless cardiac surgery has become possible as the size of cardiopulmonary bypass (CPB) circuits have decreased [2, 3]; however, blood transfusion remains an important component of neonatal cardiac surgery and postoperative management in most cardiac centers [4]. Furthermore, surgical trauma and intraoperative bleeding often necessitate blood transfusions to stabilize circulatory hemodynamics and ensure hemodynamic stability.

However, blood transfusions are not a harmless intervention. Neonates have an underdeveloped immune system and poor tolerance to allogeneic blood. Blood transfusions may trigger a series of immune reactions, including transfusion-associated acute lung injury, hemolytic reactions, and infections [5, 6, 7]. Several studies in recent years have shown that blood transfusions are associated with adverse events such as longer duration of mechanical ventilation and prolonged hospitalization after cardiac surgery in children [8, 9, 10, 11]. These events were associated with serious complications and increased hospitalization costs [8, 12, 13]. However, research on this specific population of neonates remains insufficient. Further research on the factors affecting blood transfusion and postoperative recovery is needed to improve surgical outcomes in this patient population.

This study aimed to investigate the factors influencing blood transfusion following neonatal cardiac surgery and evaluate its impact on clinical outcomes. The findings aim to provide a theoretical foundation and practical guidance for developing more evidence-based and rational blood transfusion strategies, ultimately enhancing the success rate of neonatal cardiac surgery and improving postoperative survival and quality of life.

## 2. Methods

### 2.1 Patients

**Fig. 1.**
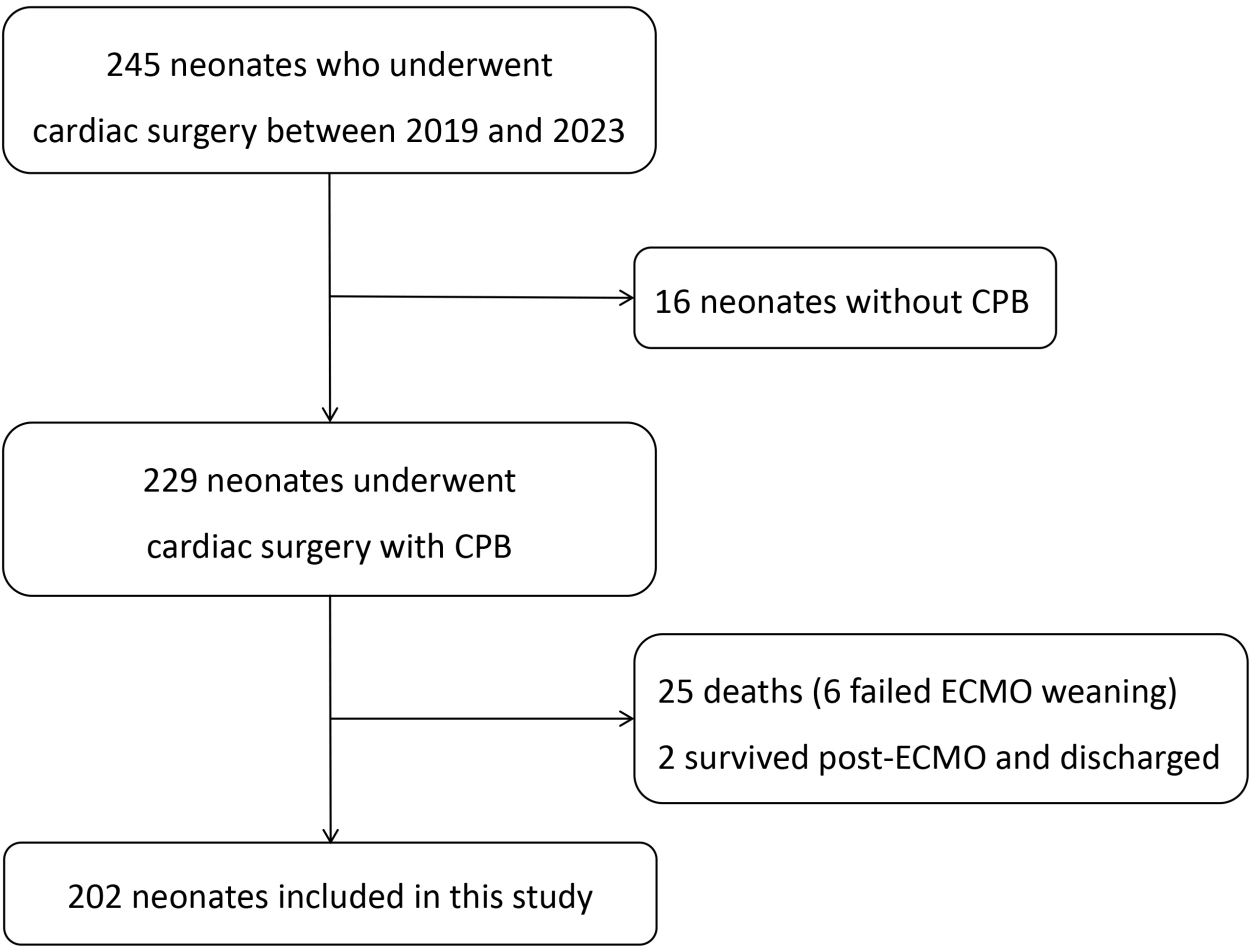
**Study flowchart**. CPB, cardiopulmonary bypass; ECMO, extracorporeal membrane oxygenation.

We retrospectively analyzed 245 neonates admitted to the intensive care unit (ICU) after cardiac surgery between January 1, 2019, and December 31, 2023, at the Beijing Anzhen Hospital. Since this study was retrospective and patient information was anonymized, informed consent was not required. 16 patients who underwent non-extracorporeal circulation surgery were excluded. Additionally, 25 patients died postoperatively, resulting in a mortality rate of 10.2%. A total of 8 patients required extracorporeal membrane oxygenation (ECMO) support after surgery, of which 2 survived and were discharged; these patients were also excluded (Fig. 1). The study protocol was approved by the Ethics Committee of the Beijing Anzhen Hospital (Number: 2024237X).

### 2.2 Variables

Demographic data, including age at surgery, weight, length, sex, and prematurity (defined as gestational age <37 weeks), were extracted for all patients from the hospital’s electronic medical record system. Detailed records of cardiac malformations and surgical procedures were maintained, and cardiac surgeries were classified using the Risk-Adjusted Classification of Congenital Heart Surgery 1 (RACHS-1) score [14].

Preoperative baseline characteristics were documented, including hemoglobin and platelet levels, the presence of cyanosis, preoperative invasive mechanical ventilation, history of cardiopulmonary resuscitation, use of inotropic agents, and administration of prostaglandin E1 (PGE1). Intraoperative data included CPB duration, aortic cross-clamp time, use of deep hypothermic circulatory arrest (DHCA), need for aortic re-cross-clamping, and delayed sternal closure. Serum lactate levels were measured at specific time points: preoperatively, at the conclusion of surgery, and 24 hours postoperatively.

According to previous studies [13, 15], the changes in the patient’s condition within 48 hours after surgery are closely related to the prognosis, therefore we recorded the blood product infusion volume (including red blood cells (RBCs), fresh frozen plasma (FFP) and platelet concentrate (PC)) and the maximum vasoactive-inotrope score (VIS) value during this period. The postoperative course was recorded, including the duration of mechanical ventilation, ICU stay and the occurrence of complications. Based on the results of a previous study [16], we defined prolonged mechanical ventilation (PMV) as greater than or equal to 96 hours after neonatal cardiac surgery. The following formula was used to calculate the VIS value [17]:

VIS = dopamine dose [µg/kg/min] + dobutamine dose [µg/kg/min] + 100 × epinephrine dose [µg/kg/min] + 10 × milrinone dose [µg/kg/min] + 10,000 × vasopressin dose [U/kg/min] + 100 × norepinephrine dose [µg/kg/min].

Data collection was independently conducted by two authors, who subsequently cross-referenced, reconciled, and validated the dataset to resolve discrepancies. Definitions for all variables were standardized to ensure consistency and reliability in data analysis.

### 2.3 Anesthesia

An individualized anesthesia management plan was developed by the anesthesiology team based on each patient’s diagnosis and clinical condition. Sevoflurane was used as the inhaled anesthetic, while sufentanil, midazolam, and rocuronium were administered intravenously. Following induction of anesthesia, standard monitoring was implemented, including central venous pressure measurement through a central venous catheter inserted in the internal jugular vein or femoral vein; systemic arterial blood pressure monitoring via catheterization of the femoral or brachial artery; urine output monitoring with an indwelling urinary catheter; and core body temperature measurement using temperature probes placed in the esophagus/nasopharynx or rectum. Heparin was administered intravenously for anticoagulation at a dosage of 3 mg/kg. Following CPB, the anticoagulant effect of heparin was neutralized by an intravenous injection of ichthyoglobulin at a dose 1.5 times the dose of heparin given in the first injection.

### 2.4 CPB Management

For neonatal CPB, a specialized neonatal tubing set was prepared and pre-filled with approximately 230 mL of pre-flush fluid, maintained at 35 °C. The circuit prime solution consisted of leukocyte-reduced RBCs, 50 mL of 20% human albumin, 110 mL of compound electrolyte solution, 15 mL of 5% sodium bicarbonate, and 1500 U of heparin sodium. CPB was initiated once the activated coagulation time (ACT) exceeded 480 seconds. A vacuum-assisted venous drainage (VAVD) device was employed to aid venous blood return. During CPB, venous oxygen saturation and hematocrit (HCT) were continuously monitored. Blood gas management was performed using an alpha steady-state approach, adjusting ventilation, oxygenation, and acid-base balance to maintain the appropriate ranges. Re-warming began after satisfactory correction of the cardiac malformation. The heart resumed beating automatically following the cessation of ascending aortic clamping. CPB was discontinued once hemodynamics stabilized and the nasopharyngeal temperature reached 37.0 °C, with a rectal temperature of 36.6 °C. Modified ultrafiltration was then performed to optimize fluid balance and enhance postoperative recovery.

### 2.5 Statistical Analysis

Normality of continuous variables was tested using the Kolmogorov-Smirnov test. If the data distribution was non-normal, it was expressed by the median (interquartile range (IQR)) and differences between groups were compared using the Mann-Whitney U test. Categorical variables were expressed as counts and percentages and compared using the chi-square test. Variables with a *p*-value < 0.1 in the univariate logistic regression were included in the forward stepwise multivariate logistic regression analysis to identify factors independently associated with PMV. A *p*-value of <0.05 was considered statistically significant. All analyses were conducted using SPSS (version 27.0, IBM SPSS Statistics, IBM Corp., Armonk, NY, USA). Data visualization was performed using R software (version 4.2.3, R Foundation for Statistical Computing, Vienna, Austria. https://www.R-project.org/) and GraphPad Prism (version 9.5.0, GraphPad Software, Boston, MA, USA. https://www.graphpad.com/).

## 3. Result

A total of 202 neonates with congenital heart disease were ultimately included in this study, with the primary diagnoses summarized in Table 1. In this cohort, the most common diagnosis was total anomalous pulmonary venous connection (TAPVC), affecting 46 (22.8%) patients, followed by transposition of the great arteries (TGA) in 34 (16.8%) patients and tetralogy of Fallot (TOF) in 27 (13.3%) patients. All patients underwent biventricular correction surgery.

**Table 1.** **Primary diagnoses**.

Primary diagnoses	N (%)
Total anomalous pulmonary venous connection	46 (22.8)
Transposition of great arteries	34 (16.8)
Tetralogy of Fallot	27 (13.3)
Pulmonary atresia	24 (11.9)
Coarctation of aorta	21 (10.4)
Pulmonary stenosis	15 (7.4)
Ventricular septal defect	13 (6.4)
Interruption of aortic arch	12 (5.9)
Others	10 (5.0)

**Fig. 2.**
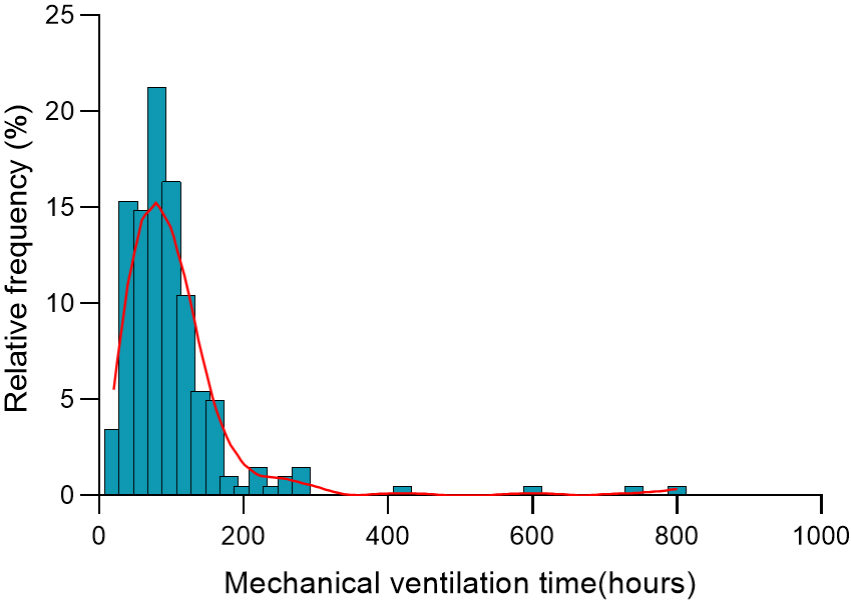
**The histogram shows mechanical ventilation time distribution in hours after neonatal cardiac surgery**. The bars represent the percentage of patients within each hour of mechanical ventilation, and the red line is the smoothed density distribution.

**Fig. 3.**
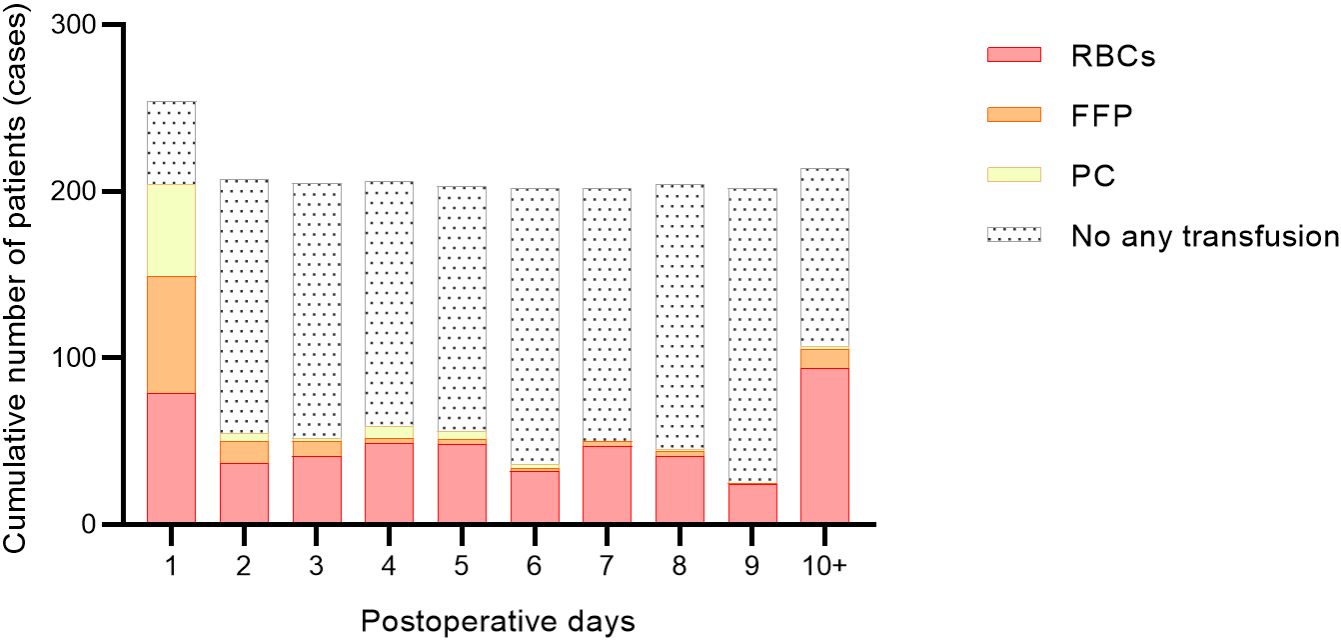
**Cumulative number of patients receiving blood product transfusions after surgery**. RBCs, red blood cells; FFP, fresh frozen plasma; PC, platelet concentrate.

The median age at surgery was 12 days (IQR, 6.00–19.00) and the median weight was 3.3 kg (IQR, 2.90–3.60) in 202 neonates, of whom 133 (65.84%) were male, 18 (8.91%) were preterm, 71 (35.15%) presented with cyanosis. In this cohort, 15 (7.43%) neonates underwent cardiopulmonary resuscitation prior to surgery, 27 (13.37%) neonates required tracheal intubation before the operation, and 14 (6.93%) cases were emergency surgeries. The median CPB time was 142.5 (IQR, 107.25–185.00) minutes, and 39 (19.31%) patients underwent DHCA. The median mechanical ventilation time (MVT) was 84.5 (IQR, 62.25–116.75) hours, and 74 (36.63%) neonates were more than or equal to 96 hours (Table 2). Fig. 2 shows the distribution frequency of MVT after cardiac surgery. Fig. 3 demonstrates the trend in the cumulative number of patients transfused with blood products after surgery.

**Table 2.** **Baseline characteristics and perioperative data of the patients (n = 202)**.

Variables		Median (IQR)/n (%)	Variables		Median (IQR)/n (%)
Pre-operative			Intra-/post-operative		
Age at admission (days)		1.00 (0.00, 12.00)	CPB time (mins)		142.50 (107.25, 185.00)
Male (n)		133 (65.84)	Cross-clamp time (mins)		78.50 (59.25, 111.00)
Weight (kg)		3.30 (2.90, 3.60)	Re-cross-clamp, n (%)		5 (2.48)
Body length (cm)		50.00 (49.00, 50.00)	DHCA, n (%)		39 (19.31)
Gestational age (weeks)		39.00 (38.00, 39.00)	MVT (hours)		84.50 (62.25, 116.75)
Age at surgery (days)		12.00 (6.00, 19.00)	MVT ≥96 hours		74 (36.63)
Prematurity (n)		18 (8.91)	Maximum VIS		10.00 (8.00, 16.00)
Cyanosis (n)		71 (35.15)	Delayed sternal closure (n)		34 (16.83)
Inotropic agents use (n)		40 (19.80)	Use of NIV (n)		70 (34.65)
PGE1 use (n)		69 (34.16)	ICU LOS (days)		9.00 (7.00, 13.00)
Resuscitation (n)		15 (7.43)	Hospital LOS (days)		15.00 (11.00, 20.00)
Emergency surgery (n)		14 (6.93)	Complications		
Tracheal intubation (n)		27 (13.37)		Peritoneal dialysis (n)	28 (13.86)
Hemoglobin (g/L)		142.00 (124.25, 162.00)		Atelectasis (n)	32 (15.84)
Platelet (×10^9^/L)		289.00 (225.25, 371.75)		Arrhythmia requiring therapy (n)	33 (16.34)
RACHS-1 (n)				Chylothorax (n)	3 (1.49)
	2	62 (30.69)		Wound infection (n)	10 (4.95)
	3	74 (36.63)		Pleural effusion requiring drainage (n)	11 (5.45)
	4	66 (32.67)		Peritoneal effusion requiring drainage (n)	40 (19.80)
				Reintubation (n)	10 (4.95)

PGE1, prostaglandin E1; RACHS-1, Risk-Adjusted Classification of Congenital Heart Surgery 1; IQR, interquartile range; CPB, cardiopulmonary bypass; DHCA, deep hypothermic circulatory arrest; MVT, mechanical ventilation time; VIS, vasoactive-inotrope score; NIV, non-invasive ventilation; ICU, intensive care unit; LOS, length of stay. Data were present as n (%), or median (interquartile range) according to variable category.

**Fig. 4.**
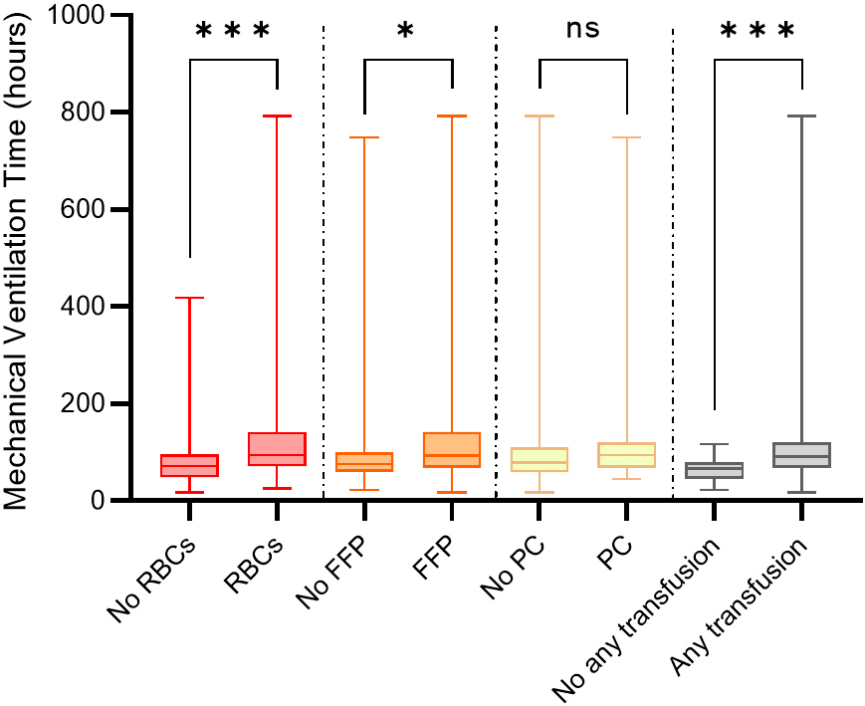
**Difference in mechanical ventilation time between different blood product transfusion and no transfusion**. RBCs, red blood cells; FFP, fresh frozen plasma; PC, platelet concentrate. *, *p* < 0.05, ***, *p* < 0.001. ns, *p* > 0.05.

As shown in Table 3, within 48 hours after surgery, 101 (50.00%) patients were transfused with RBCs, 76 (37.62%) with FFP, 56 (27.72%) with PC, and only 42 (20.79%) were not transfused with any blood products. There was also a significant difference (*p* < 0.001) in MVT between patients receiving a transfusion (91.00, IQR, 68.00–120.00 hours) and those without any transfusion (66.50, IQR, 46.00–79.00 hours; Fig. 4).

**Table 3.** **Postoperative differences in MVT by blood product transfusion within 48 hours**.

Variables		MVT (hours)	*p*-value
RBCs			<0.001
	No (n = 101, 50.00%)	71.00 (49.00, 94.00)	
	Yes (n = 101, 50.00%)	94.00 (71.00, 141.00)	
FFP			0.016
	No (n = 126, 62.38%)	76.00 (60.25, 98.75)	
	Yes (n = 76, 37.62%)	93.00 (68.00, 141.25)	
PC			0.069
	No (n = 146, 72.28%)	79.00 (59.25, 106.50)	
	Yes (n = 56, 27.72%)	93.50 (68.00, 120.00)	
Any transfusion			<0.001
	No (n = 42, 20.79%)	66.50 (46.00, 79.00)	
	Yes (n = 160, 79.21%)	91.00 (68.00, 120.00)	

MVT, mechanical ventilation time; RBCs, red blood cells; FFP, fresh frozen plasma; PC, platelet concentrate. Data were present as n (%), or median (interquartile range) according to variable category.

**Fig. 5.**
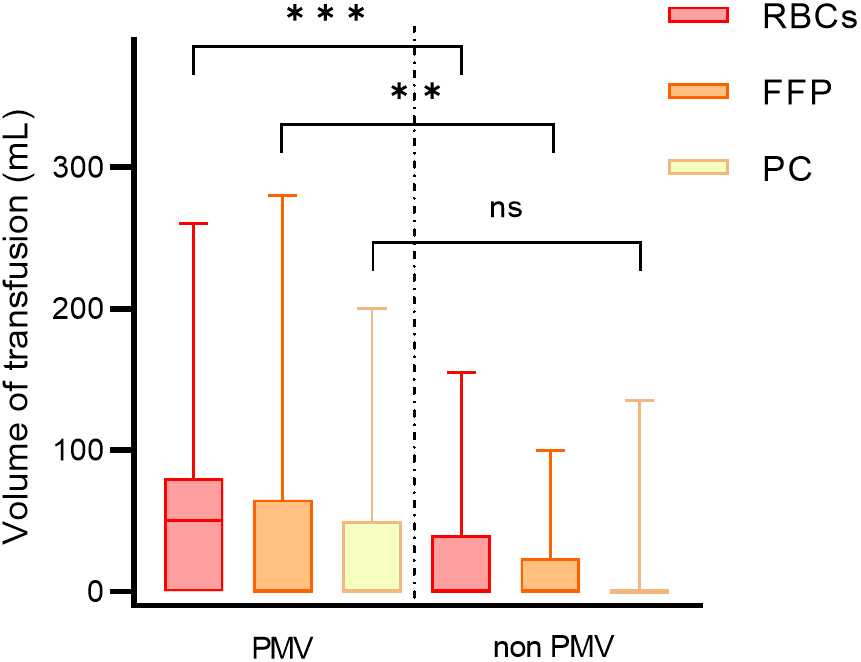
**Blood transfusion volume of patients in different groups**. PMV, prolonged mechanical ventilation; RBCs, red blood cells; FFP, fresh frozen plasma; PC, platelet concentrate. ***, *p* < 0.001; **, *p* < 0.01. ns, *p* > 0.05.

The differences in perioperative data between PMV patients and non-PMV patients are shown in Table 4. The incidence of PMV was 36.63% (74/202). Neonates requiring prolonged mechanical ventilation exhibited lower body weight, reduced gestational age, increased prematurity, decreased preoperative hemoglobin levels, and longer times on CPB (all *p*-values < 0.05). The volume of RBCs and FFP transfusions within 48 hours after surgery was also higher in these patients (*p* < 0.001, *p* = 0.004, respectively). However, the transfusion volume of PC did not show a significant difference (*p* = 0.065) (Fig. 5). Additionally, the postoperative recovery process was more challenging in patients with PMV, as evidenced by an increased use of non-invasive ventilation (NIV) (50.00% vs. 25.78%), longer ICU length of stay (LOS) (14.00 [IQR, 11.00–19.75] vs. 8.00 [6.00–10.00] days), and extended hospital LOS (18.00 [IQR, 15.00–24.00] vs. 13.00 [IQR, 10.00–17.00] days) (all *p*-values < 0.001).

**Table 4.** **Baseline characteristics and perioperative data in PMV and non-PMV patients**.

Variables		Non-PMV (n = 128)	PMV (n = 74)	*p*-value
Pre-operative				
	Age at admission (days)	2.00 (0.00, 13.00)	1.00 (0.00, 10.00)	0.524
	Male (n)	86 (67.19)	47 (63.51)	0.596
	Weight (kg)	3.40 (3.10, 3.70)	3.00 (2.80, 3.50)	<0.001
	Body length (cm)	50.00 (49.00, 50.00)	50.00 (49.00, 50.00)	0.292
	Gestational age (weeks)	39.00 (38.00, 39.00)	38.00 (37.00, 39.00)	0.002
	Age at surgery (days)	12.00 (6.75, 18.00)	12.50 (6.00, 20.00)	0.673
	Prematurity (n)	5 (3.91)	13 (17.57)	0.001
	Cyanosis (n)	41 (32.03)	30 (40.54)	0.222
	Inotropic agents use (n)	22 (17.19)	18 (24.32)	0.220
	PGE1 use (n)	45 (35.16)	24 (32.43)	0.694
	Resuscitation (n)	7 (5.47)	8 (10.81)	0.163
	Emergency surgery (n)	9 (7.03)	5 (6.76)	0.941
	Tracheal intubation (n)	16 (12.50)	11 (14.86)	0.643
	Hemoglobin (g/L)	146.00 (128.00, 168.25)	134.50 (117.25, 151.00)	0.003
	Platelet (×10^9^/L)	299.00 (233.50, 375.00)	267.00 (215.50, 353.75)	0.225
	Preoperative lactate (mmol/L)	1.50 (1.00, 2.00)	1.70 (1.10, 2.60)	0.061
RACHS-1 (n)				0.180
	2	45 (35.16)	17 (22.97)	
	3	45 (35.16)	29 (39.19)	
	4	38 (29.69)	28 (37.84)	
Intra-/post-operative				
	CPB time (mins)	138.00 (105.00, 177.25)	165.00 (121.25, 204.00)	0.023
	Cross-clamp time (mins)	75.50 (55.00, 106.00)	88.00 (64.00, 114.00)	0.093
	Re-cross-clamp, n (%)	2 (1.56)	3 (4.05)	0.530
	DHCA, n (%)	22 (17.19)	17 (22.97)	0.316
	Lactate on ICU arrival (mmol/L)	1.95 (1.30, 3.30)	3.25 (2.20, 4.27)	<0.001
	Lactate on 24 hours after operation (mmol/L)	2.00 (1.50, 2.70)	2.40 (1.80, 3.58)	0.027
	Maximum VIS	10.00 (7.38, 15.00)	12.00 (8.62, 18.00)	0.067
	Use of NIV (n)	33 (25.78)	37 (50.00)	<0.001
	ICU LOS (days)	8.00 (6.00, 10.00)	14.00 (11.00, 19.75)	<0.001
	Hospital LOS (days)	13.00 (10.00, 17.00)	18.00 (15.00, 24.00)	<0.001
	0–48 h post-surgery RBCs (mL)	0.00 (0.00, 40.00)	50.00 (0.00, 80.00)	<0.001
	0–48 h post-surgery FFP (mL)	0.00 (0.00, 21.25)	0.00 (0.00, 65.00)	0.004
	0–48 h post-surgery PC (mL)	0.00 (0.00, 0.00)	0.00 (0.00, 50.00)	0.065

PMV, prolonged mechanical ventilation; PGE1, prostaglandin E1; RACHS-1, Risk-Adjusted Classification of Congenital Heart Surgery 1; CPB, cardiopulmonary bypass; DHCA, deep hypothermic circulatory arrest; VIS, vasoactive-inotrope score; NIV, non-invasive ventilation; ICU, intensive care unit; LOS, length of stay; RBCs, red blood cells; FFP, fresh frozen plasma; PC, platelet concentrate. Data were present as n (%), or median (interquartile range) according to variable category.

**Fig. 6.**
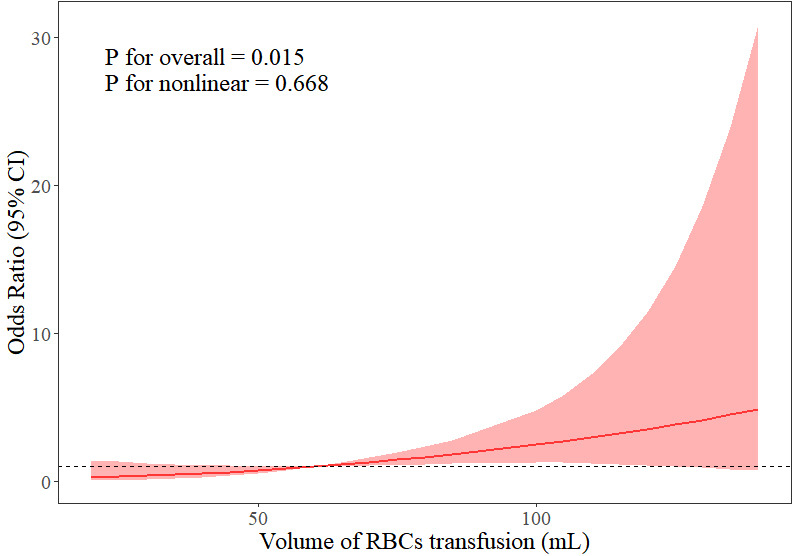
**Relationship between duration of mechanical ventilation and volume of RBCs transfusion within 48 hours after surgery**. RBCs, red blood cells; CI, confidence interval.

Multivariate logistic regression analysis (Table 5) showed lower body weight (odds ratio (OR) = 0.38, 95% confidence interval (CI): 0.20–0.74, *p* = 0.005), lower preoperative hemoglobin level (OR = 0.99, 95% CI: 0.97–0.99, *p* = 0.041), RACHS-1 score of grade 4 (OR = 2.56, 95% CI: 1.04–6.27, *p* = 0.040), transfusion of RBCs within 48 hours after surgery (OR = 2.02, 95% CI: 1.02–4.00, *p* = 0.043), and transfusion of FFP (OR = 1.98, 95% CI: 1.02–3.85, *p* = 0.043) were independent risk factors for PMV. Fig. 6 shows a restricted cubic spline (RCS) plot after controlling for the confounders of body weight, hemoglobin, RACHS-1, age at surgery, and sex. The RCS plot suggests a dose-response relationship between RBCs transfusion volume and PMV, i.e., the higher the transfusion volume, the greater the risk of PMV (*p* = 0.015). This relationship is more likely to be linear than complex and nonlinear (*p* = 0.668). However, after controlling for these confounding variables, there was no relationship between the volume of FFP transfusion and PMV (**Supplementary Fig. 1**).

**Table 5.** **Univariate and multivariate logistic regression for postoperative PMV**.

		Univariate analysis		Multivariate analysis	
		OR (95% CI)	*p*-value	OR (95% CI)	*p*-value
Prematurity					
	No	Reference		Reference	
	Yes	5.24 (1.79∼15.38)	0.003	2.52 (0.75∼8.43)	0.135
	Weight (kg)	0.34 (0.19∼0.61)	<0.001	0.38 (0.20∼0.74)	0.005
	Preoperative Hb (g/L)	0.98 (0.97∼0.99)	0.004	0.99 (0.97∼0.99)	0.041
RACHS-1					
	2	Reference		Reference	
	3	1.71 (0.82∼3.53)	0.150	1.64 (0.63∼4.05)	0.324
	4	1.95 (0.93∼4.09)	0.077	2.56 (1.04∼6.27)	0.040
CPB time (min)		1.01 (1.01∼1.01)	0.032	1.00 (1.00∼1.01)	0.513
RBCs					
	No	Reference		Reference	
	Yes	2.86 (1.58∼5.20)	<0.001	2.02 (1.02∼4.00)	0.043
FFP					
	No	Reference		Reference	
	Yes	1.90 (1.06∼3.43)	0.032	1.98 (1.02∼3.85)	0.043

PMV, prolonged mechanical ventilation; Hb, hemoglobin; RACHS-1, Risk-Adjusted Classification of Congenital Heart Surgery 1; CPB, cardiopulmonary bypass; RBCs, red blood cells; FFP, fresh frozen plasma; OR, odds ratio; CI, confidence interval. The RBCs and FFPs counted were infused within 48 hours postoperatively.

Differences in complication rates were evaluated between patients with and without RBC transfusions, with statistical significant differences in the incidence of peritoneal dialysis, arrhythmias, peritoneal effusions and PMV (Table 6).

**Table 6.** **Differences in complication rates between patients with and without RBCs transfusion**.

Main complications	No-RBCs	RBCs	*p*-value
	(n = 101)	(n = 101)	
Peritoneal dialysis	7 (6.93)	21 (20.79)	0.004
Atelectasis	11 (10.89)	21 (20.79)	0.054
Arrhythmia requiring therapy	11 (10.89)	22 (21.78)	0.036
Chylothorax	2 (1.98)	1 (0.99)	1.000
Wound infection	3 (2.97)	7 (6.93)	0.194
Pleural effusion requiring drainage	3 (2.97)	8 (7.92)	0.121
Peritoneal effusion requiring drainage	10 (9.90)	30 (29.70)	<0.001
Reintubation	4 (3.96)	6 (5.94)	0.517
PMV	25 (24.75)	49 (48.51)	<0.001

RBCs, red blood cells; PMV, prolonged mechanical ventilation.

## 4. Discussion

This study systematically analyzed the association between postoperative blood transfusion and PMV in neonates undergoing cardiac surgery. The results showed that transfusion of RBCs and FFP within 48 hours after surgery were independent risk factors for PMV. In addition, there was a dose-response relationship between the volume of RBCs transfused and the risk of PMV, with a significant increase in the risk of PMV for each threshold increase in the volume of RBCs transfusion (*p* = 0.015). Other risk factors included low body weight, low preoperative hemoglobin level, and high RACHS-1 score (grade 4). These findings highlight the potential negative impact of blood transfusion in postoperative respiratory management and provide an important basis for optimizing neonatal blood transfusion strategies.

The association between blood transfusion and PMV may be mediated through multiple mechanisms. First, transfusion of allogeneic blood products activates systemic inflammatory responses and releases pro-inflammatory cytokines (e.g., interleukin 6, tumor necrosis factor-α), leading to pulmonary capillary leakage and alveolar edema, which in turn exacerbates respiratory insufficiency [18]. Second, storage-damaged erythrocytes release free hemoglobin and iron ions, which damage pulmonary vascular endothelial cells through oxidative stress and reduce pulmonary compliance [19, 20]. In addition, transfusion-associated immunomodulation may suppress the immune function of children [21] and increase the risk of nosocomial infections [22], which are a common trigger of PMV. Higher rates of NIV utilization and longer ICU stays in PMV patients in this study further support the hypothesis of transfusion-related lung injury.

Using RCS analysis, this study revealed for the first time in neonates, a linear dose-effect relationship between RBCs infusion volume and PMV (*p* nonlinear = 0.668). This result is consistent with studies in adult cardiac surgery [23, 24, 25, 26]. However, neonates are more sensitive to transfusion volume because of their small blood volume and metabolic fragility. Possible explanations for the increased sensitivity of neonates to large infusion volumes is that transfusions dilute coagulation factors [27], increasing the risk of postoperative bleeding and the need for a re-sternotomy, which indirectly prolongs mechanical ventilation. High volume loads also increase the cardiopulmonary burden, which is especially pronounced in low-body-weight neonates [28, 29, 30].

Although FFP infusion was independently associated with the risk of PMV (OR = 1.98), the mechanism is different from that of RBCs. FFP is enriched in coagulation factors and complement components, which may exacerbate lung injury through microthrombosis and complement activation [31, 32, 33]. However, the present study found no dose-effect relationship between FFP infusion volume and PMV (**Supplementary Fig. 1**), suggesting that the risk may be more related to infusion decision-making (e.g., using infusions when bleeding tendency is evident) rather than infusion volume alone. This result differs from some studies [25, 34] and may reflect the heterogeneity of the indications for FFP use in neonates, which needs to be further validated in the future in conjunction with dynamic monitoring of coagulation function.

In addition, multivariate analysis showed that higher body weight (OR = 0.38, 95% CI: 0.20–0.74) and higher preoperative hemoglobin level (OR = 0.99, 95% CI: 0.97–0.99) were independent protective factors against PMV, with the risk decreasing with increasing body weight and hemoglobin level. Neonates with low body weight have limited cardiopulmonary reserve and are more dependent on blood transfusion to maintain oxygen supply [35], resulting in a vicious cycle of “low body weight-transfusion-PMV”. Preoperative anemia may exacerbate postoperative tissue hypoxia and induce multiple organ dysfunction by decreasing the efficiency of oxygen delivery [36]. RACHS-1 score grade 4 (OR = 2.56) reflects surgical complexity, and its association with PMV may be due to more complex surgical procedures, higher risk of postoperative infections, and more significant systemic inflammatory response [37, 38]. These risk factors, along with blood transfusions lead to the development of PMV.

This study provides several interventions to reduce PMV. Modified ultrafiltration and autologous blood transfusion were used intraoperatively to reduce the need for allogeneic blood. In neonates with low body weight and high RACHS scores, anemia was corrected preoperatively, and lung-protective ventilation strategies were initiated early postoperatively. In addition, promotion of bloodless pre-filled CPB lines may further reduce neonatal blood exposure.

This study has the following limitations. First, because the data were derived from a retrospective analysis, there may have been unmeasured confounders that could have influenced the association between transfusions and PMV. Second, despite the inclusion of 202 neonates, this sample size was insufficient for certain subgroup analyses (e.g., FFP transfusion volume stratification) may have reduced the statistical efficacy and limited in-depth exploration of these subgroups. Third, the specific indications and decision-making process for blood transfusion were not documented in detail in this study, which may affect the interpretation and generalization of the results. Fourth, all data were obtained from a single center, which may have a selection bias and limit the external validity of the results. Future studies should further elucidate the causal relationship between blood transfusion and PMV through a multicenter prospective design and evaluate the clinical benefits of restrictive transfusion strategies.

## 5. Conclusion

Blood transfusions after neonatal cardiac surgery is an important controllable risk factor for PMV, and its risk increases linearly with the increase of RBC infusions. Optimizing transfusion strategies, utilizing blood protection technologies, and stratifying the management of high-risk patients, is expected to reduce the incidence of PMV and improve the prognosis of neonates requiring cardiac surgery.

## Availability of Data and Materials

The datasets used and/or analysed during the current study are available from the corresponding author on reasonable request.

## Supplementary Material

Supplementary material associated with this article can be found, in the online version, at https://doi.org/10.31083/RCM36566.
