# Supplementary figures and images for: Blood Transfusions are Associated With Prolonged Mechanical Ventilation Following Cardiac Surgery in Neonates
Source: Rev Cardiovasc Med. 2025 Jun 26;26(6):36566. doi: 10.31083/RCM36566 (PMC12230815; doi:10.31083/RCM36566)

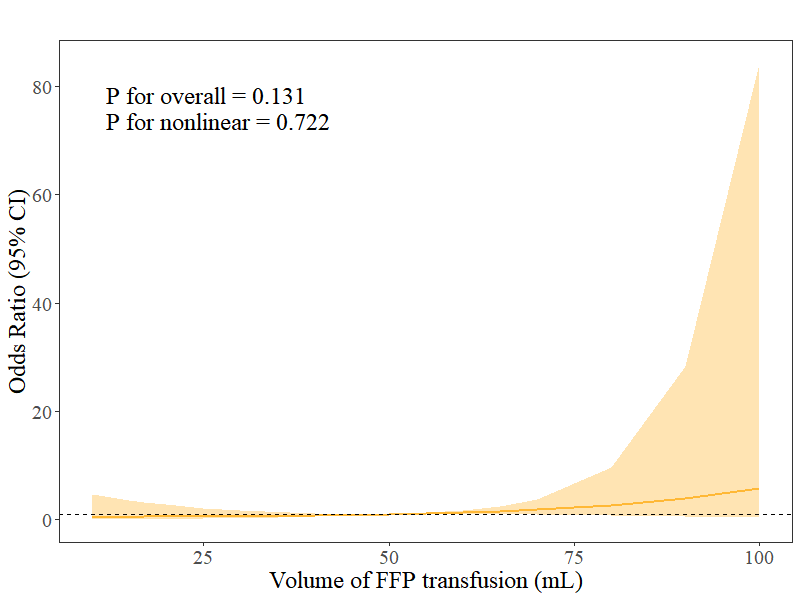

Supplement: Supplementary file 1 [file 2153-8174-26-6-36566-s1.tiff]
